# Radiation Inhibits Interleukin-12 Production via Inhibition of C-Rel through the Interleukin-6/ Signal Transducer and Activator of Transcription 3 Signaling Pathway in Dendritic Cells

**DOI:** 10.1371/journal.pone.0146463

**Published:** 2016-01-08

**Authors:** Eun-Jung Lee, Seo Jin Lee, Ji-Hye Kim, Kyoung-Jin Kim, Seung-Hyun Yang, Keun-Yeong Jeong, Jinsil Seong

**Affiliations:** Department of Radiation Oncology, Yonsei Cancer Center, Yonsei University College of Medicine, Seoul, 120–752, Republic of Korea; Center for Cancer Research, National Cancer Institute, UNITED STATES

## Abstract

Radiotherapy (RT) is a potent anti-tumor modality. However, unwanted effects including increased recurrence and metastasis that involve factors such as cytokines, which induce complex molecular mechanisms, have also been reported. In a previous study, we showed that interleukin (IL)-12 and radiotherapy combination treatment suppressed tumor growth and metastasis in a hepatoma mouse model. In this study, we investigated the mechanism underlying the IL-12 anti-tumor effect during radiotherapy. In tumor-bearing mice, irradiation decreased IL-12 expression in the tumors and spleens. However, a number of dendritic cells infiltrated into the tumors in which IL-12 expression did not decrease. To further study the underlying detailed mechanism for this decrease in IL-12, LPS-stimulated bone marrow–derived dendritic cells (BMDCs) were irradiated, and then IL-12– and IL-6–associated molecules were examined in irradiated tumors and BMDCs. Irradiation resulted in IL-12 suppression and IL-6 increase. IL-6 and signal transducer and activator of transcription 3 (STAT3) inhibitors restored the irradiation-induced IL-12 decrease via suppression of C-Rel activation. Taken together, our study suggests that irradiation-induced IL-6 can decrease IL-12 production through the inhibition of C-Rel phosphorylation by the IL-6/STAT3 signaling pathway.

## Introduction

Radiation (RT) is a potent anti-tumor modality used to treat various cancers, including glioma, breast, colon, lung, liver, head, and neck cancers. Radiation can bring about immune cell recruitment to tumors through the high-mobility group protein B1 (HMGB1) released by dying cells, calreticulin caused by cellular stress response, and releases of mediators such as inflammatory cytokines and chemokines [1–3]. In particular, RT strongly stimulates interleukin (IL)-6 production by various cells, including immune cells, epithelial cells, and fibroblasts in radiotherapy [4, 5]. IL-6 is closely involved in growth and metastasis regulation in cancers as well as in the host immune defense mechanism for foreign pathogens [6, 7]. Classically, IL-6 activates cells through binding to the membrane-bound IL-6 receptor (IL-6R) followed by homodimerization of a 130 kDa signal-transducing element (gp130) molecules, which facilitates the activation of downstream signal cascades [8]. Even in the absence of IL-6R, gp130 expressing cells can be activated by the complex of IL-6 and soluble IL-6R and this process has been called trans-signaling [9]. The sIL-6R binds to IL-6 with similar affinity as the membrane bound IL-6R does. A soluble form of the IL-6R (sIL-6R) can be generated by limited proteolysis of the membrane bound receptor [10, 11] and by translation of a differentially spliced mRNA [12, 13]. IL-6 directly activates the signal transducers and activators of transcription (STAT) factors, including STAT1 and STAT3, via the Janus kinases (JAK) [14]. In cancers, the main mechanisms involved in STAT3 activation are IL-6 autocrine synthesis and IL-6 paracrine activation from the stroma and infiltrating inflammatory-associated immune cells [15]. Particularly, STAT3 expression in the tumor microenvironment can modulate IL-12 and IL-23 secretion by immune cells such as dendritic cells (DCs) and macrophages [16].

IL-12 is the most critical factor in the Th1 immune response that is induced by foreign pathogen–stimulated DCs [17]. IL-12 is considered to be a useful candidate for anti-tumor therapies, as its expression at the tumor site could attract activated lymphocytes such as CD8^+^ T cells [18]. In addition, IL-12 is crucial for the development of the peritumoral stroma required for the acceptance of tumor-migrating T cells [19]. It has also been reported that IL-12p70–producing DC vaccine elicits CD8^+^ cytotoxic T cell–mediated immunity [18].

We previously reported the anti-tumor effect of RT combination treatment and IL-12 expressed through both mesenchymal stem cells and armed oncolytic adenovirus in a murine hepatic cancer model [20, 21]. However, the detailed molecular mechanism of the IL-12 regulation of RT was not demonstrated. Therefore, the present study was designed to demonstrate the underlying mechanism of the IL-12/RT combination treatment and IL-12 regulation in DCs by radiation.

## Materials and Methods

### Reagents

Antibodies against STAT3, phosphorylated STAT3 (p-STAT3), C-Rel, and p-C-Rel were purchased from Bioss (Woburn, MA). WST-1 cell proliferation assay kit was obtained from Roche Diagnostics (Mannheim, Germany). FITC Anti-mouse B7-1 (Clone 16-10A1), PE Anti-mouse B7-2 (Clone GL1), PE Anti-Mouse IL-12 p40/p70 (Clone C15.6) and APC Anti-mouse CD11c (Clone HL3) antibodies were purchased from BD Pharmingen (San Diego, CA). IL-6 and IL-12 ELISA kit were obtained from R&D System Inc. (Minneapolis, MN). Anti-IL-6 (ab6672) and anti-CD11c (ab33484) antibodies for immunofluorescent (IF) staining were purchased from abcam (San Francisco, CA). Anti-IL-12 (NB600-1443) antibody for IF staining was purchased from Novusbio (Littleton, CO). Mouse sIL-6R alpha Simple Step ELISA Kit (ab203360) was obtained from abcam (Cambridge, MA). Cell Fixation/Permeabilization Kits (BD554715) for intracellular cytokine analysis was purchased from BD Bioscience (San Jose, CA). Purified Rat Anti-mouse IL-6 antibody (clone MP5-20F3) was obtained from BD Biosciences. STAT3 inhibitor VI, S31-201 was purchased from Santa Cruz Biotechnology.

### Ethics statement

This study was carried out in strict accordance with the recommendations in the Laboratory Animals Welfare Act, the Guide for the Care and Use of Laboratory Animals of the National Institutes of Health. The protocol was approved by the Committee on the Ethics of Animal Experiments of the Yonsei University Health System (Permit Number: 2012–0177). All animal surgery was performed after euthanasia using CO_2_, and all efforts were made to minimize suffering.

### Animal experimental design and X-ray irradiation

Five male C3H/HeN mice, 6–7 weeks old (Central Lab, Japan), were used for each experimental group. HCa-1 [22, 23] and MIH-2 [24, 25] murine hepatoma cells (1 × 10^6^ cells) in 100 μL PBS were injected intramuscularly into the right thighs of the mice, and tumors were irradiated with 10 Gy when tumors reached 8 mm in diameter in a single fraction using an X-Rad 320 irradiator (Precision X-ray, North Branford, CT, USA). Mice were placed at 69 cm from the radiation source (SSD) for the treatment at a dose rate of 150 cGy/min with 300 kVp X-rays, using 12.5 mA and a X-ray beam filter consisting of 2.0 mm Al. At 1, 3 and 7 day after 10 Gy radiation, tumors were collected to evaluate expression of IL-6, IL-12 and DCs. All procedure of animal research was provided in accordance with the Laboratory Animals Welfare Act, the Guide for the Care and Use of Laboratory Animals and the Guidelines and Policies for Rodent experiment provided by the Animal Care and Use Committee of Yonsei University Health System. At the end of the experiment, the experimental mice were euthanized using carbon dioxide (CO_2_) before the tumor reached the maximum allowable size.

### DC purification from bone marrow cultures

Bone marrow–derived DCs (BMDCs) were generated from a culture of bone marrow cells from mouse tibial and femoral bones, as described previously [26, 27]. The cells (1 × 10^6^ cells/mL) were seeded onto a six-well culture plate in complete Roswell Park Memorial Institute medium (RPMI)-1640 supplemented with recombinant murine granulocyte macrophage colony-stimulating factor (GM-CSF; 10 ng/mL) and IL-4 (10 ng/mL). The culture medium was changed every 2 days to remove granulocytes. Loosely adherent clustering cells were used as immature DCs for this study.

### Purified BMDCs irradiation

BMDCs were further seeded at 1 × 10^6^/mL/well onto 24-well plates in the presence of 100 ng/mL lipopolysaccharide (LPS) and then irradiated with 5 or 10 Gy. Supernatants were collected from each well 24 and 72 h after the irradiation for cytokine measurement.

### Cell viability assay

BMDCs were seeded in 96 well flat bottom plates. 24 hours after seeding, cells were irradiated with 10 Gy. After 72 days, a WST-1 assay was performed as recommended by the manufacturer (Roche Diagnostics, Mannheim, Germany). Then, the conversion of WST-1 was quantified in an enzyme-linked immunosorbent assay (ELISA)-reader at 450 nm.

### B7-1 and B7-2 expression in DCs

LPS-stimulated BMDCs were irradiated with 5 or 10 Gy and incubated for 48 h. These cells were resuspended in phosphate-buffered saline (PBS) and stained with FITC-labeled B7-1 and PE-labeled B7-2 antibodies at a final concentration of 1 μg/mL for 15 min at 4°C. Cells were then washed twice and immediately analyzed using a FACS Verse flow cytometer (BD Bioscience, San Joes, CA).

### Intracellular staining for IL-12 detection

Surface staining for DCs detection used antibodies to APC-labeled CD11c (BD Bioscience) Following surface staining, intracellular IL-12 was stained as recommended by the manufacturer using Cell Fixation/Permeabilization Kits (BD Bioscience). Briefly, surface stained BMDCs were fixed and permeabilized for 40 min in fixation/ permeabilization buffer. After washing with 1xPerm/Wash buffer, those were stained with PE-conjugated IL-12 antibody for 30 min on ice/ light protected. Cells were then washed twice with 1xPerm/Wash buffer and immediately analyzed using a FACS Verse flow cytometer. Samples were analyzed with a FlowJo (Tree Star Inc, San Carlo, CA)

### Immunofluorescence

Differential immunofluorescence analyses were performed on 5-μm cryostat sections. The following antibodies were used: Gout anti-IL-12 (1:100 dilution; Novusbio, NB600-1443), Hamster anti-CD11c (1:100 dilution: Abcam, ab33484), rabbit anti-IL-6 (1:100 dilution; Abcam, ab6672), and Alexa 488 anti-goat IgG, Alexa 488 anti-Hamster IgG and Alexa 594 anti-rabbit IgG (Life Technologies). The slides were mounted in SlowFade Gold Antifade Reagent with 4′, 6-diamidino-2-phenylindole (Life Technologies).

### Cytokine production quantitation

DCs were resuspended in media containing GM-CSF and IL-4 and incubated for the indicated times in the presence of 100 ng/mL LPS. At the end of the incubation, supernatants were decanted, non-adherent cells were removed by centrifugation, and the cell-free supernatants were stored at −70°C until use. IL-6 and IL-12 were measured using commercially-available enzyme-linked immunosorbent assays (ELISA Quantikine kits, R&D System Inc.) following the manufacturer’s instructions.

### Quantitative real-time PCR analysis

Total RNA was extracted from DCs using TRIzol (Invitrogen), and cDNA was synthesized from 1μg of total RNA by reverse transcription (Qiagen). Real-time PCR analysis was performed using a Step One Plus-Real Time system (Applied Biosytems, Tokyo, Japan) to measure SYBR Green (Applied Biosystems). Relative amounts of mRNA were normalized to GAPDH mRNA levels within each sample. The primer sequences used were: IL-12p40: Forward 5’-AAC CAT CTC CTG GTT TGC CA-3’ and Reverse 5’-CGG GAG TCC AGT CCA CCT C-3’, IL-12p35: Forward 5’- CCC TTG CAT CTG GCG TCT ACA CTG CTG C -3’ and Reverse 5’—AGG AGG GCA AGG GTG GCC AAA AAG AGG A-3’.

### Statistical analysis

The data are presented as means ± SD, and statistical significance between groups was assessed by unpaired two-sided *t*-tests. All experiments were performed at least three times. Differences of *P* < 0.05 (*) and *P* < 0.01 (**) were considered to be statistically significant.

## Results

### Irradiation inhibited IL-12 production by DCs

**Fig 1:**
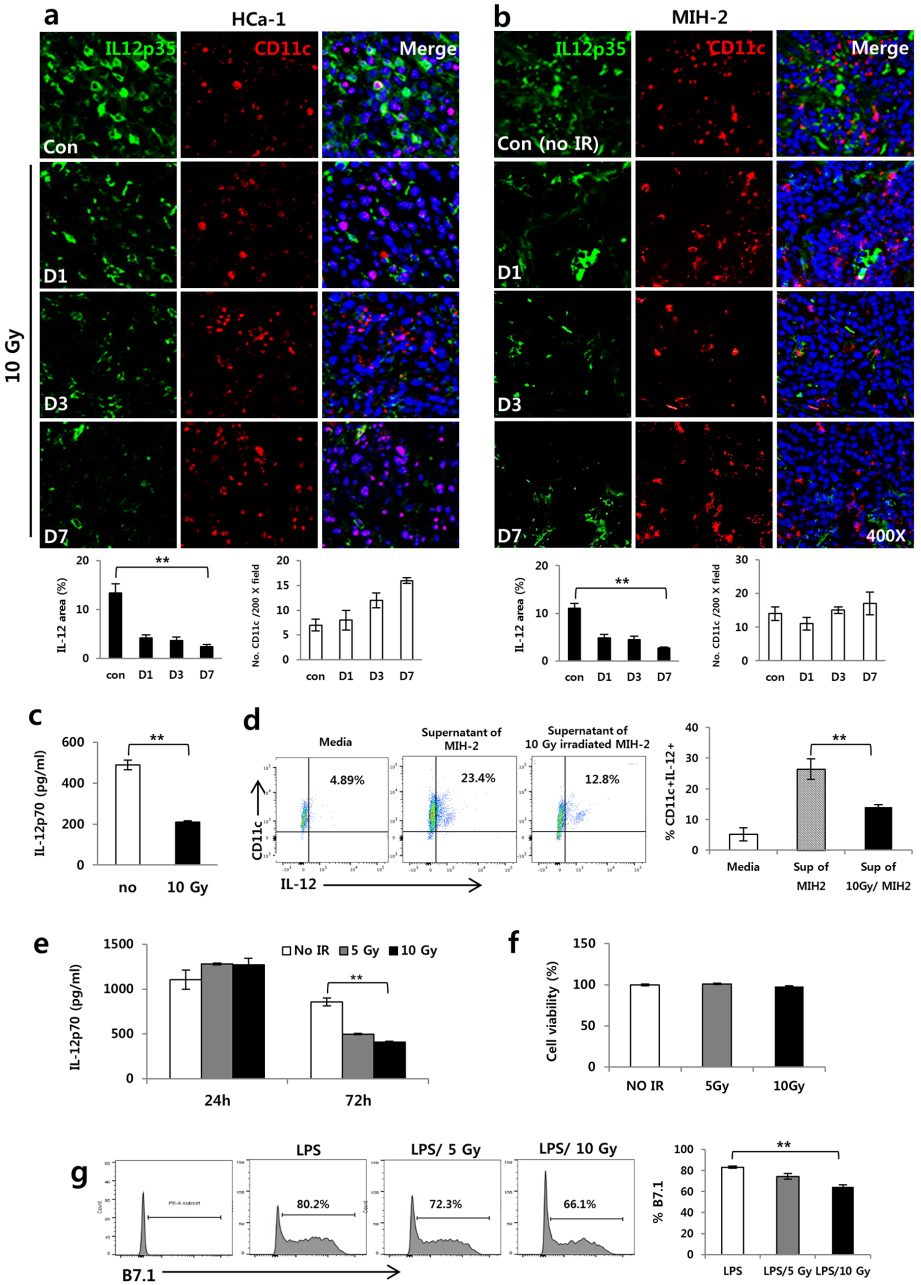
Irradiation inhibited IL-12 expression in tumors of hepatoma-bearing mice *in vivo* and DCs *in vitro*. HCa-1 and MIH-2 cells were injected intramuscularly into the right thighs of the mice, and tumors were irradiated with 10 Gy of radiation. Shown are IL-12 and DC (CD11c^+^) expressions at 1, 3, and 7 days after irradiation in (a) HCa-1 and (b) MIH-2 tumors and (c) IL-12 expression in the spleens of HCa-1 bearing mice (** *P* < 0.01). (d) IL-12 expression in BMDCs incubated with supernatant of MIH-2 tumor cells or supernatant of 10 Gy irradiating MIH-2 tumor cells. (** *P* < 0.01). DCs were differentiated from bone marrow of C3H/HeN normal mice (BMDCs). These were incubated for 72 h in supernatant of MIH-2 tumor cells with or without 10 Gy irradiation. IL-12 was intracellular stained with IL-12 antibody and analyzed by FACS. BMDCs also were stimulated with LPS (100 ng/mL) to allow maturation and irradiated with 10 Gy of radiation. (e) IL-12 expression (** *P* < 0.01), (f) cell viability, and (g) B7.1 expression in LPS stimulated BMDCs receiving 10 Gy radiation. (* *P* < 0.05). Data are from three independent experiments with five mice per group.

To determine whether irradiation regulates IL-12 expression in tumors, HCa-1 and MIH-2 cells (1 × 10^6^ cells) were injected into the right thighs of C3H/HeN mice, and tumors were irradiated with 10 Gy when the tumor volume reached about 250 mm^3^. HCa-1 is a rapidly-growing hepatoma that is resistant to radiation, while MIH-2 is a slow-growing hepatoma that is sensitive to radiation in relative comparison to HCa-1. IL-12 is mainly produced by DCs in response to tumor-associated antigens or during the release of damage-associated molecular patterns (DAMPs) such as HMGB1, HSP, and calreticulin in the tumor microenvironment [3]. Therefore, tumors were co-stained with IL-12 and CD11c antibodies to investigate the presence of DCs and IL-12 release from DCs at 1, 3, and 7 days after irradiation in tumors. As shown in Fig 1A and 1B, irradiation dramatically decreased IL-12 expression over time after irradiation, while the levels of DCs as CD11c-positive cells were not significantly changed by irradiation in both tumors. In addition, IL-12 secretion from the spleens of the irradiating tumor group was decreased compared to the control tumor group (Fig 1C). The tumor associated-soluble factors such as HMGB1, growth factors, chemokines and cytokines could contribute to the tumor growth through the regulation and alteration of tumor microenvironment. Therefore, to investigate possible tumor-derived soluble factors induced by irradiation that contributed to the decrease of IL-12 in DCs, DCs were isolated from mouse bone marrow cells (BMDCs) that were differentiated in media containing IL-4 (10 ng/mL) and GM-CSF (10 ng/mL) for 6 days and then, these were incubated for 72 h in supernatants of MIH-2 cell irradiated with or without 10 Gy. BMDCs were analyzed by flow cytometry (FACS) after intracellular staining for IL-12 detection. As a result, BMDCs of 10 Gy irradiated-MIH-2 culture supernatant showed lower IL-12 level than BMDCs of non-irradiated MIH-2 culture supernatant (Fig 1D). Therefore, to investigate whether irradiation regulates IL-12 production by DCs, BMDCs were cultured for 24 and 72 h after irradiation in media containing GM-CSF and IL-4 with the presence of 100 ng/mL LPS, which was used to allow DCs to mature. IL-12 was measured in the culture supernatant. The resulting IL-12 secretion increased slightly after 24 h of irradiation; however, it decreased significantly after 72 h of irradiation (Fig 1E). DC viability was not affected by irradiation (Fig 1F). Also, to measure the activation of DCs, the expression of B7.1 as a co-stimulating molecule was assessed by FACS. BMDCs were subcultured in GM-CSF–and IL-4–supplemented media for 48 h in 24-well plates after irradiation in the presence of LPS. As shown in Fig 1G, irradiation led to a decrease in B7.1 expression when compared to LPS treatment alone. These results suggest that irradiation could suppress IL-12 expression in tumors and tumor microenvironments, including DCs.

### Irradiation-induced IL-6 suppressed IL-12 production in tumors and DCs

**Fig 2:**
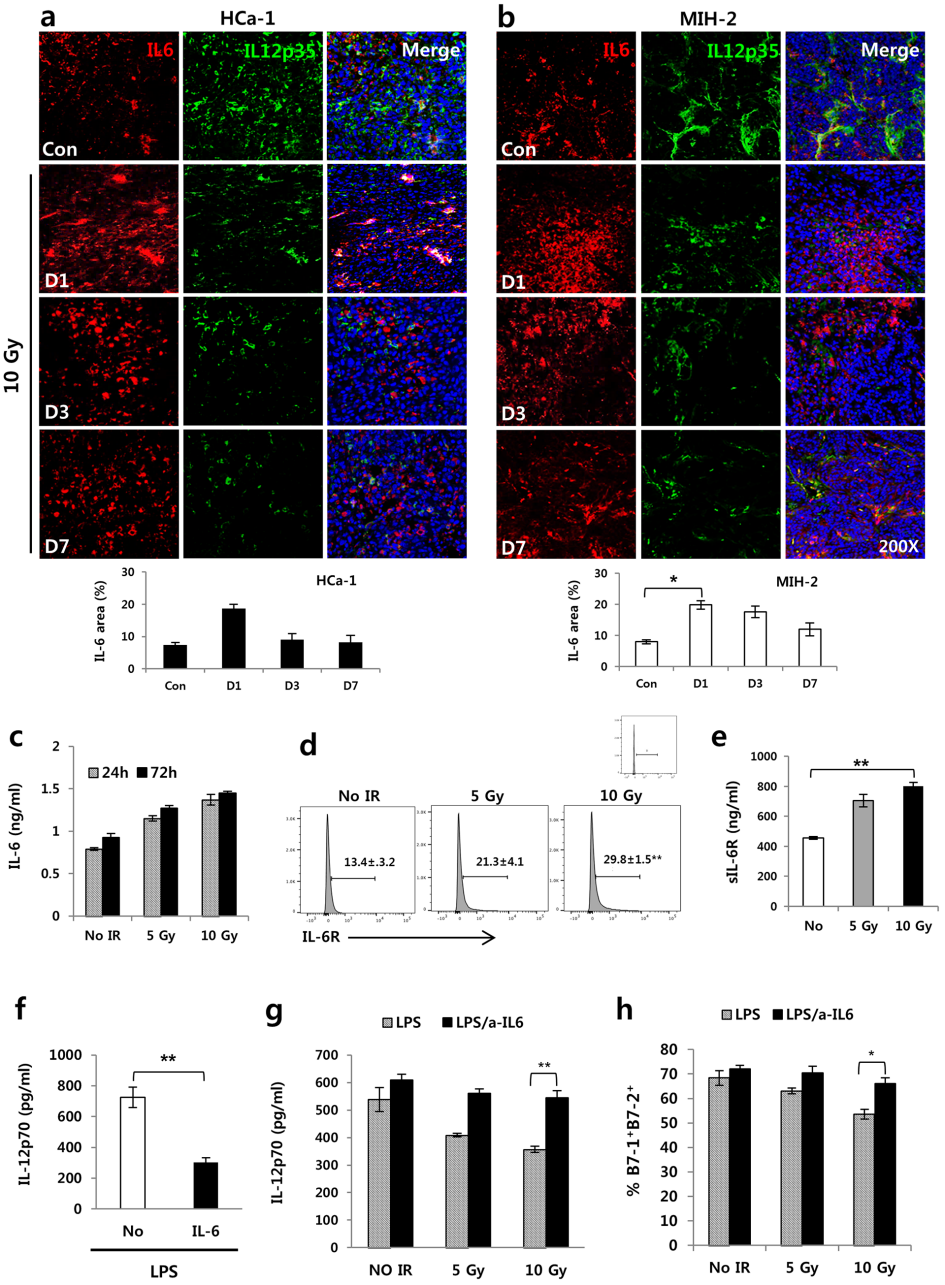
Irradiation-induced IL-6 suppressed IL-12 expression in tumors of hepatoma-bearing mice *in vivo* and DCs *in vitro*. IL-6 and IL-12 were measured at 1, 3, and 7 days after 10 Gy irradiation of tumors. Shown are the expressions of IL-6 and IL-12 in (a) HCa-1 and (b) MIH-2 tumors (* *P* < 0.05). BMDCs were prepared from bone marrow of mice and stimulated with LPS. (c) IL-6 expression analyzed at the indicated times by ELISA in irradiated or non-irradiated BMDCs *in vitro*. (d) IL-6R (** *P* < 0.01, *vs* No IR) and (e) sIL6R (** *P* < 0.01) were evaluated at 2 day after 10 Gy irradiation to BMDCs by FACS and ELISA, respectively. BMDCs were treated without or with IL-6 (20 ng/mL) plus LPS. (f) Down regulation of IL-12 production by IL-6 treatment in BMDCs (** *P* < 0.01). BMDCs were stimulated with LPS for activation and cultured with or without anti-IL-6 antibody (final concentration of 500 ng/mL) during the indicated times after irradiation *in vitro*. IL-6 and IL-12 were analyzed by ELISA. (g) Recovery of IL-12 production and (h) B7-1/ B7-2 expression using an anti-IL-6 antibody in irradiated-BMDCs stimulated with LPS *in vitro*. (** *P* < 0.01, * *P* < 0.05). Data are from three independent experiments.

Hoentjen et al. showed that IL-10 induced–STAT3 regulates IL-12 production in LPS-stimulated DCs [28]. STAT3 is activated in response to IL-6 [29], and irradiation can substantially induce IL-6 production. Therefore, we investigated whether irradiation-induced IL-6 could regulate IL-12 production in tumors. As shown in Fig 2A and 2B, IL-6 expression was upregulated in both types of 10 Gy irradiated–tumors compared to the control tumors, whereas IL-12 expression decreased as opposed to IL-6 expression. In addition, 10 Gy irradiated BMDCs exhibited higher expression of IL-6R and sIL-6R (Fig 2C and 2D) than no-irradiation. Additionally, IL-6 treatment to BMDCs decreased IL-12 secretion from BMDCs compared to no treatment (Fig 2E). We also investigated whether irradiation regulated IL-6 production in DCs. BMDCs were incubated in media containing GM-CSF, IL-4, and LPS for 24 and 72 h after 10 Gy irradiation. As a result, IL-6 secretion in the culture supernatant from DCs was highly increased by irradiation at both 24 and 72 h (Fig 2F). Considering that IL-6 secreted by irradiation from DCs and tumor cells may affect DCs through autocrine and paracrine signaling, we investigated whether IL-6 could hinder IL-12 production in DCs. LPS-stimulated BMDCs were immediately treated with or without an anti-IL-6 antibody after irradiation. As a result, IL-6 inhibition rescued IL-12 production from the irradiation-induced inhibition (Fig 2G). Additionally, irradiation suppressed the expression of B7-1 and B7-2 in a radiation dose dependent manner compared to LPS treatment alone, and anti-IL-6–antibody treatment rescued B7-1 and B7-2 expression from the irradiation-induced inhibition (Fig 2H). Therefore, these results indicate that irradiation-induced IL-6 could suppress IL-12 production by DCs.

### Irradiation-induced STAT3 activation inhibited IL-12 secretion from DCs

**Fig 3:**
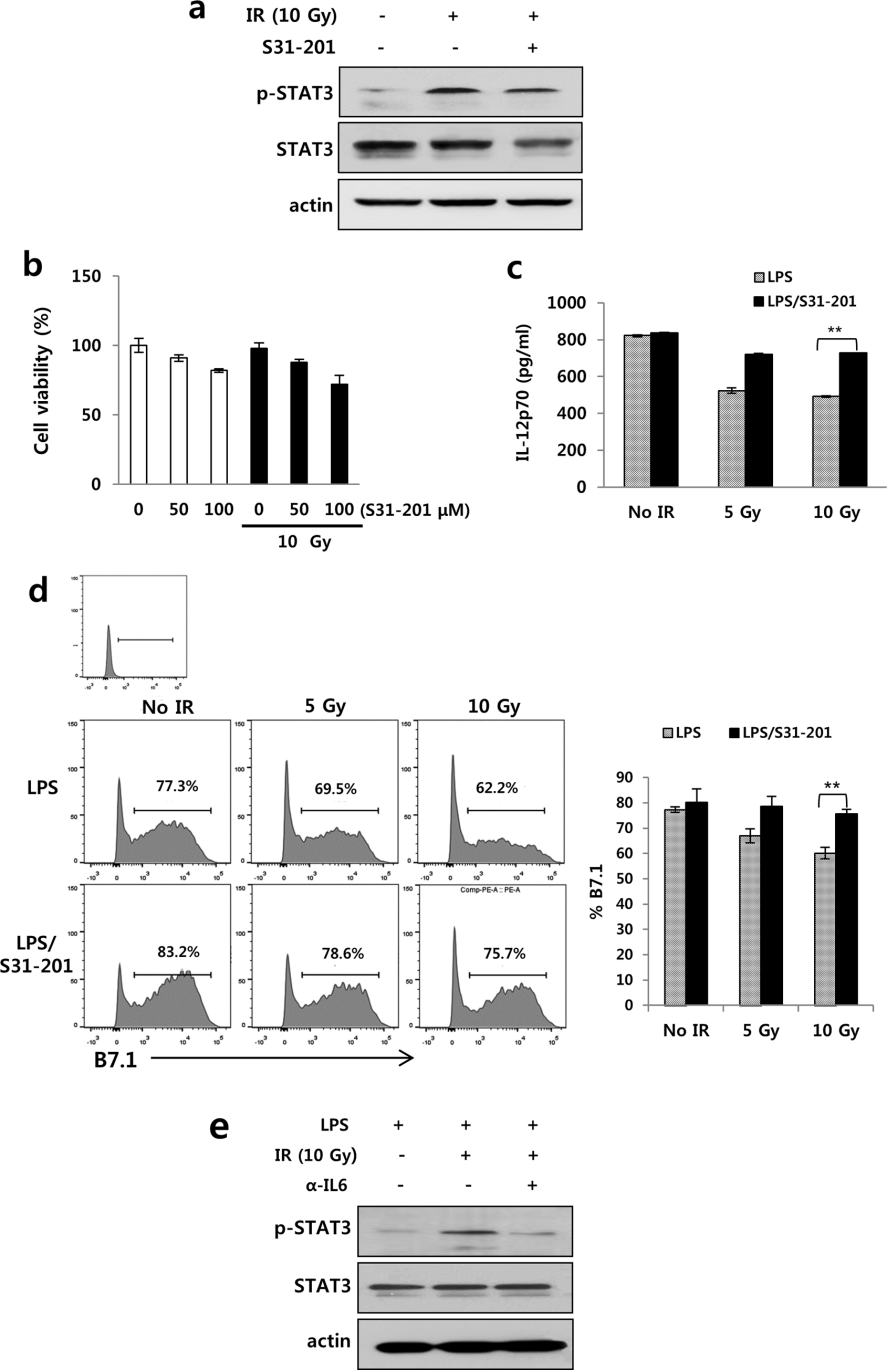
STAT3 activation suppressed IL-12 expression in irradiated-BMDCs. LPS-stimulated BMDCs were pretreated with S31-201, STAT3 inhibitor (final concentration of 50 μM) for 24 h prior to 10 Gy irradiation, and then p-STAT3 and STAT3 were analyzed by western blotting at 1 h after irradiation. (a) STAT3 expression and (b) cell viability in S31-201 treated BMDCs. LPS-stimulated BMDCs were treated with or without S31-201 followed by 10 Gy irradiation. The expression of IL-12 and B7-1 were evaluated at 3 day after irradiation by FACS. (c) Recovery of IL-12 and (d) B7-1 expression by S31-201 in LPS-stimulated BMDCs receiving 10 Gy radiation (** *P* < 0.01). (e) Expression of p-STAT3 suppressed by anti-IL-6 antibody treatment in LPS-stimulated BMDCs receiving 10 Gy radiation. Data are from three independent experiments.

STAT3 was originally identified as an acute-phase response factor that responds to stimulation by IL-6 [30]. As STAT3 can also be activated by radiation via regulation of other molecules such as survivin as well as IL-6 induction [31], STAT3 inhibitor was used to test whether the STAT3 activation was involved in the irradiation-induced decrease in IL-12. S31-201, STAT3 inhibitor, was added to LPS-stimulated BMDCs receiving 10 Gy radiation, and were incubated for the indicated times. As shown in Fig 3A and 3B, S31-201 inhibited phosphorylation of stat3, but it doesn’t significantly affect cell viability. STAT3 inhibitor treatment restored the expression of IL-12 and B7-1 that had been inhibited by radiation (Fig 3C and 3D). Anti-IL-6 antibody treatment inhibited STAT3 phosphorylation in LPS-stimulated BMDCs receiving 10 Gy irradiation (Fig 3E). These results suggest that irradiation induced IL-6 secretion, which activated STAT3, and finally could inhibit IL-12 production in DCs.

### IL6-induced STAT3 activation interfered with IL-12 synthesis through suppression of C-Rel phosphorylation in irradiated-DCs

**Fig 4:**
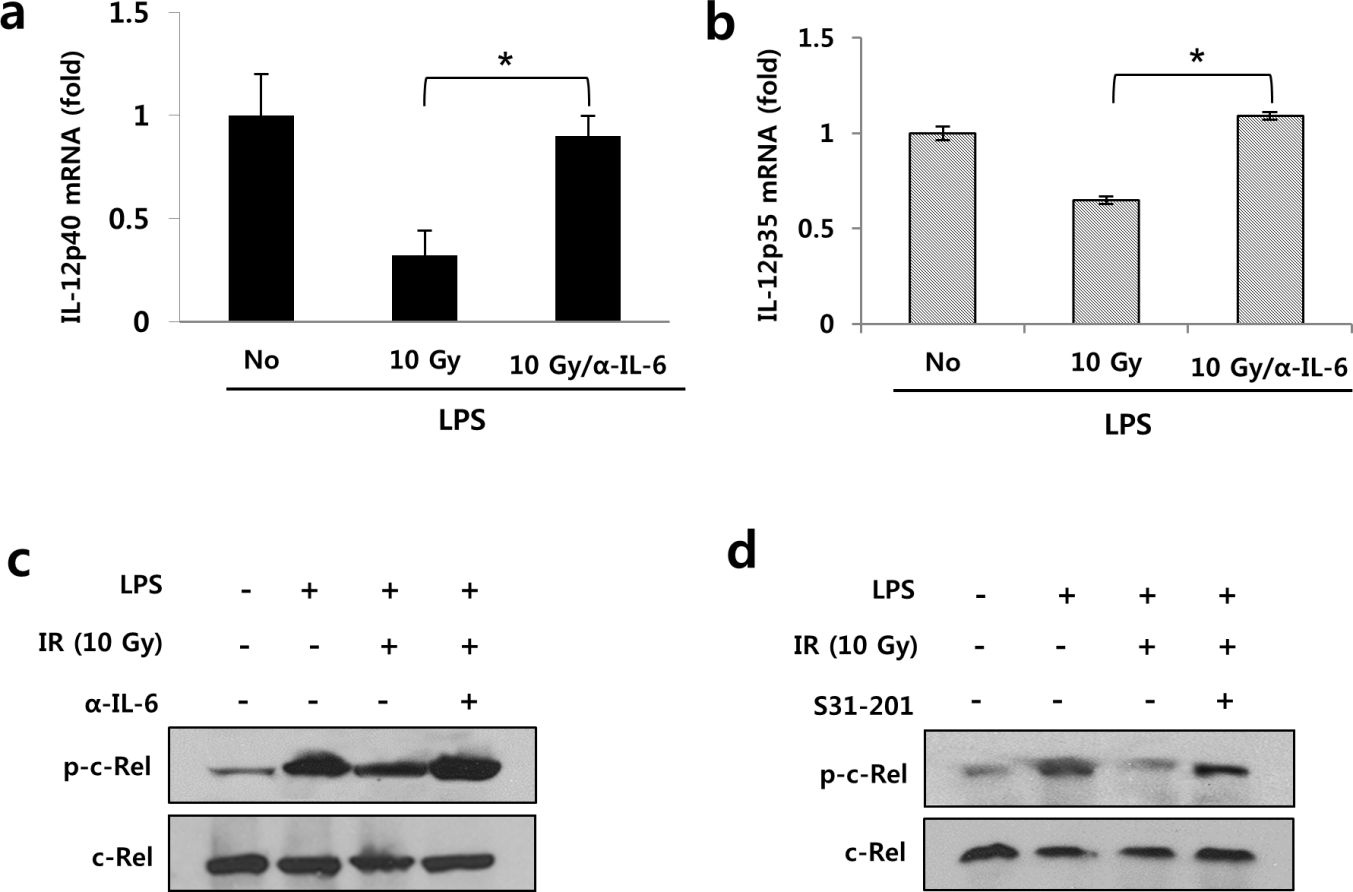
IL-6/STAT3 signaling interfered with IL-12 synthesis via the blocking of C-Rel phosphorylation in irradiated BMDCs. BMDCs were stimulated with LPS for maturation and cultured with anti-IL-6 antibody (500 ng/mL) or STAT3 inhibitor (50 μM) for 16 h after irradiation. The IL-12 mRNA level was evaluated using quantitative real-time PCR. (a) Upregulation of IL-12p40 mRNA and (b) IL-12p35 mRNA by anti-IL-6 antibody in LPS-stimulated BMDCs receiving 10 Gy radiation (* *P* < 0.05). (c) Recovery of radiation-suppressed C-Rel phosphorylation by IL-6 blocking and (d) STAT3 inhibition in LPS-stimulated BMDCs receiving 10 Gy radiation. Data are from three independent experiments.

**Fig 5:**
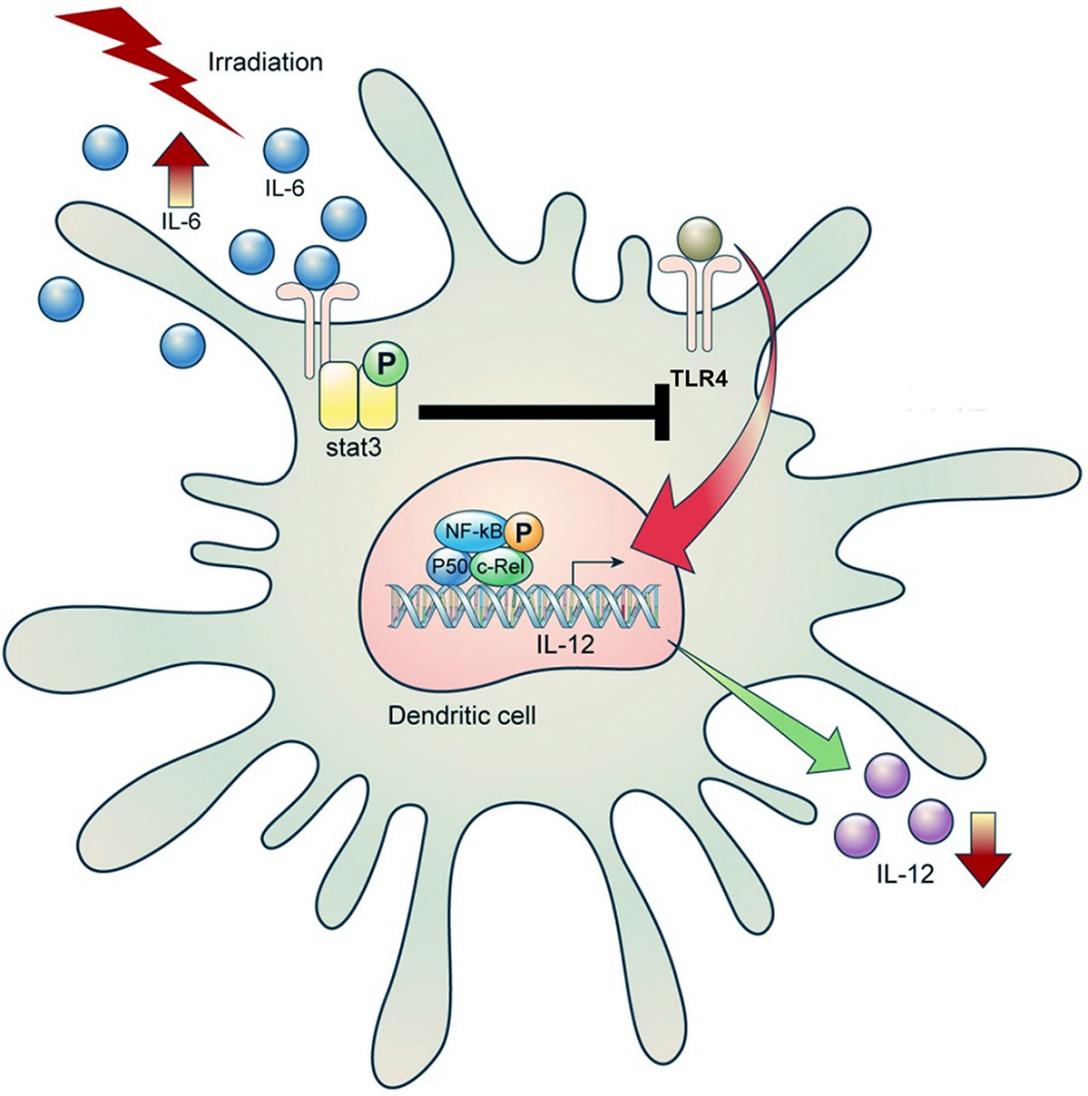
Schematic model of the signaling pathway involved in IL-12 inhibition by irradiation in tumor-associated DCs.

To determine the underlying mechanism by which IL-6 inhibited IL-12 production in DCs, we examined the levels of IL-12 mRNA in LPS-stimulated DCs with or without irradiation. As shown in Fig 4A and 4B, inhibition of IL-6 increased the levels of IL-12p40 as well as IL-12p35 mRNA. According to a previous report, NF-kappa B (NF-kB) family members, including p50/p65 and p50/C-Rel, act as transcriptional factors in IL-12p40 activation [32]. In particular, STAT3 can affect IL-12 production via C-Rel [28]. Therefore, we investigated the expression of C-Rel in LPS-stimulated DCs. LPS-stimulated BMDCs were incubated with or without irradiation for 16 h and collected for western blotting. C-Rel expression was increased by the anti-IL-6 antibody and STAT3 inhibitor in 10 Gy-irradiated BMDCs (Fig 4C and 4D). Thus, as shown in Fig 5, irradiation could interfere with IL-12 synthesis via inhibition of C-Rel activation through the IL-6/STAT 3 pathway in DCs.

## Discussion

We previously showed the anti-tumor effect of an IL-12/RT combination treatment [20, 21]. However, its detailed molecular mechanism had not been demonstrated. Therefore, the present study was designed to define the role of IL-12 in radiotherapy and its underlying mechanism. We showed that irradiation inhibited IL-12 expression in HCa-1 and MIH-2 tumors and spleens of tumor-bearing mice. However, the number of DCs that mainly secrete IL-12 was more an increase than a decrease in both types of irradiated tumors (HCa-1 and MIH-2) compared to the control tumors. The HCa-1 and MIH-2 tumor cells that were used in this study differ in their sensitivity to radiation and potential for tumorigenesis [33]. Therefore, these results suggest that the observed immune responses, including cytokine regulation and immune cell infiltration by radiation, may have occurred regardless of the radio-sensitivity of the tumors. Many previous studies suggest that radiotherapy can lead to immunogenic cell death via recruitment of immune cells such as DCs, macrophages, and lymphocytes into the tumors by the release of radiation-induced DAMPs including HMGB1, HSP, and calreticulin [34, 35]. Indeed, the induction of HMGB1 in tumors was caused by 10-Gy irradiation (data not shown), which might have triggered maturation and infiltration of DCs via Toll like receptor 4 (TLR4) in this study. In spite of an increase in DCs recruitment, IL-12 expression was diminished in irradiated tumors. It has been reported that although DCs are resistant to radiation-induced apoptosis, radiation inhibits IL-12 secretion from mature DCs as a consequence of immune response suppression via impairing the priming ability of T cells [36]. However, the underlying mechanism by which radiation can downregulate IL-12 remains unclear. Accordingly, we investigated the molecular mechanism involved in the suppression of IL-12 expression by irradiation in DCs. In current study, irradiation slightly induced IL-12 expression in BMDCs 24 h after irradiation. In contrast, at 72 h after irradiation, the irradiation significantly suppressed IL-12 secretion by LPS-treated BMDCs, as well as B7.1 and B7.2 expression. These results indicate that IL-12 production could be affected by radiation-stimulated molecular factors in later responses. Accordingly, to identify the mechanism regulating IL-12 in irradiated-BMDCs, we investigated IL-6/STAT3 signaling, as STAT3 activation can disrupt IL-12 production and STAT3 is a downstream signaling molecule of the IL-6 receptor [37]. It has been reported that STAT3 signaling regulates the IL-12/IL-23 balance, which directs the transcriptional activation of the IL-23/p19 gene and inhibits IL-12/p35 gene expression in tumor-associated DCs [16]. Moreover, Radiation strongly enhances IL-6, which inhibits radiation-induced apoptosis [4]. Radiation also can induce cellular senescence, which can increase IL-6 release [38]. Indeed, 10 Gy irradiation elevated expression of β-galactosidase; senescence marker in tumors (data not shown). Accordingly, RT-induced cellular senescence might contribute to secretion of IL-6 in this study. IL-6 signal cascade is triggered by IL-6/IL-6R complex via membrane-bound receptor and a soluble form of the IL-6R, which is frequently associated with inflammatory diseases and increased risk of cancers [15]. Cellular activation by IL-6 requires binding to IL-6 receptor and resulting recruitment of two gp130. This initiates phosphorylation of gp130-associated Janus kinases (JAKs), which stimulates their phosphorylation via binding of STAT1/STAT3 to gp130 [39]. In the present study, 10 Gy irradiation enhanced expression of cell membrane bounded IL-6R and soluble IL-6R as well as IL-6 production, which might suppress IL-12 synthesis in BMDCs. Interestingly, treatment with an anti-IL-6 antibody and a STAT3 inhibitor rescued IL-12 expression in irradiated-BMDCs. These results suggest that the IL-6/STAT3 signaling pathway is closely related to the downregulation of IL-12 by radiation in irradiated BMDCs. Radiation could also activate both IL-6 signaling pathways of the classic signaling via membrane binding receptor and trans-signaling via soluble receptor. Given that NF-kBp50/C-Rel transcription factor can regulate IL-12/p40 gene expression [32], we further investigated NF-kB signaling to better understand the molecular mechanism involved in IL-12 suppression by the IL-6/STAT3 signaling in DCs. In addition, Hoentjen et al. showed that upregulation of the IL-12/p40 gene in IL-10 deficient mice is due to enhanced C-Rel recruitment to the IL-12p40 promoter in the absence of activated STAT3 [28]. Thus, activated-STAT3 inhibits IL-12 production through the interruption of C-Rel recruitment to the IL-12p40 promoter. IL-12 plays a crucial role in regulating both innate and adaptive immune responses and can be a potent anti-tumor agent that can be used in combination with other cytokines with anti-tumor activities [40]. In our study, irradiation strongly stimulated the phosphorylation of STAT3, which inhibited C-Rel phosphorylation in LPS-stimulated BMDCs. Additionally, irradiation-induced inhibition of C-Rel phosphorylation was rescued by anti-IL-6 antibody and STAT3 inhibitor. Thus, as shown in Fig 5, irradiation-induced inhibition of C-Rel phosphorylation involved the IL-6/STAT3 signaling pathway in maturated DCs, which resulted in the suppression of IL-12 synthesis.

In conclusion, the present study is the first to report that RT inhibits IL-12 through the IL-6/STAT3 signaling pathway in tumors and tumor-associated DCs. Our results imply that IL-12 treatment may be a useful therapeutic modality for cancer patients receiving RT.

## Abbreviations

RT: Radiotherapy
IL: interleukin
STAT3: signal transducer and activator of transcription 3
HCC: hepatocellular carcinoma
BMDCs: bone marrow derived dendritic cells
CD: cluster of differentiation
LPS: lipopolysaccharide
